# Infection with AV-SUR2A protects H9C2 cells against metabolic stress: A mechanism of SUR2A-mediated cytoprotection independent from the K_ATP_ channel activity

**DOI:** 10.1016/j.bbamcr.2010.01.018

**Published:** 2010-03

**Authors:** Qingyou Du, Sofija Jovanović, Andriy Sukhodub, Aleksandar Jovanović

**Affiliations:** Division of Medical Sciences, Centre for Cardiovascular and Lung Biology, Ninewells Hospital and Medical School, University of Dundee, UK

**Keywords:** SUR2A, ATP, K_ATP_ channel, Cardioprotection, Ischaemia, Heart

## Abstract

Transgenic mice overexpressing SUR2A, a subunit of ATP-sensitive K^+^ (K_ATP_) channels, acquire resistance to myocardial ischaemia. However, the mechanism of SUR2A-mediated cytoprotection is yet to be fully understood. Adenoviral SUR2A construct (AV-SUR2A) increased SUR2A expression, number of K_ATP_ channels and subsarcolemmal ATP in glycolysis-sensitive manner in H9C2 cells. It also increased K^+^ current in response to chemical hypoxia, partially preserved subsarcolemmal ATP and increased cell survival. Kir6.2AFA, a mutant form of Kir6.2 with largely decreased K^+^ conductance, abolished the effect of SUR2A on K^+^ current, did not affect SUR2A-induced increase in subsarcolemmal ATP and partially inhibited SUR2A-mediated cytoprotection. Infection with 193gly-M-LDH, an inactive mutant of muscle lactate dehydrogenase, abolished the effect of SUR2A on K^+^ current, subsarcolemmal ATP and cell survival; the effect of 193gly-M-LDH on cell survival was significantly more pronounced than those of Kir6.2AFA. We conclude that AV-SUR2A increases resistance to metabolic stress in H9C2 cells by increasing the number of sarcolemmal K_ATP_ channels and subsarcolemmal ATP.

## 1 Introduction

Cardiac sarcolemmal ATP-sensitive K^+^ (K_ATP_) channels are gated by intracellular ATP and are viewed as a link between cellular metabolism and membrane excitability. These channels are closed under physiological conditions and are opened only during ischaemia, which protect the myocardium against ischaemic damage. Structurally, sarcolemmal K_ATP_ channels *in vivo* are composed of an inward rectifier, Kir6.2 and Kir6.1, SUR2A, an ATP-binding protein, and accessory proteins that are glycolytic and ATP-producing enzymes [reviewed in 1]. Recently, it has been shown that cardiomyocytes from transgenic mice overexpressing K_ATP_ channel regulatory subunit, SUR2A, acquire resistance against hypoxia and other types of metabolic stresses. The mechanism of SUR2A-mediated cardioprotection seems to be associated with increased numbers of sarcolemmal K_ATP_ channels, earlier activation of K_ATP_ channels during stress, shortening of the action membrane potential and consequent decrease in Ca^2+^ influx [2]. It should be, however, said that in some recent studies a mechanism of cytoprotection afforded by K_ATP_ channels independent from the channel activity was reported. More specifically, it has been suggested that enzymes that are physically associated with K_ATP_ channel subunits regulate subsarcolemmal/intracellular ATP levels, which, in turn, promotes cellular survival under metabolic stress [3-5]. It is therefore possible that SUR2A-mediated cardioprotection has a component in addition to the increased channel activity.

Therefore, we have undertaken this research to elucidate the channel-dependent and channel-independent mechanisms of SUR2A-mediated cytoprotection. It has been shown that rat heart embryonic H9C2 cells are good experimental model to study SUR2A, K_ATP_ channels and cardioprotection [6]. As an example, these cells have been used to uncover the effect of increased SUR2A expression on cellular response to metabolic stress [7,8], which was shown to correspond to adult hearts exposed to hypoxia [2]. Here, we have generated adenovirus containing gene encoding SUR2A and tested the effect that this construct has on survival of H9C2 cells exposed to severe metabolic stress. We have elucidated the mechanism underlying SUR2A-mediated cytoprotection and found out that there is more to the cardioprotection by SUR2A than previously thought.

## 2 Methods

### 2.1 H9C2 cells and viral constructs

H9C2 cell rat embryonic heart H9c2 cells (ECACC, Salisbury, UK) were cultured in a tissue flask (at 5% CO_2_) containing Dulbecco's modified Eagle's medium supplemented with 10% fetal calf serum and 2 mM glutamine. For electrophysiological experiments, the cells were plated on a 35 × 10-mm culture dish containing 25-mm glass cover-slips. The cells were cultured in incubators (Galaxy, oxygen control model, RS Biotech, Irvine, UK). For the experiments H9C2 cells were infected with adenoviral constructs containing either green fluorescent protein (GFP, gift from C. Sunderland, University of Dundee; cells infected with GFP have served as control cells in this study), gly193-M-LDH (a catalytically inactive mutant of M-LDH, [9], Kir6.2, or Kir6.2AFA (a mutant form of Kir6.2 where the pore GFG was mutated into AFA leading to largely reduced K^+^ conductance, [10]). When intracellular and subsarcolemmal ATP levels were measured cells were infected with adenovirus containing luciferase and annexin VI-luciferase genes respectively. All these adenoviruses were generated and used as described in details in [4,5]. The recombinant SUR2A adenovirus (AV-SUR2A) was generated using the AdEasy XL Adenoviral Vector System (Stratagene). SUR2A gene was cloned into a shuttle vector pShuttle-CMV by PCR using the following primers containing restrict enzyme sites Bgl II/Xho I, sense, 5′-GCAGATCT GGC AGG CTG TTG GTA GCT CA-3′, antisense, 5′-GCCTCGAG CTA CTT GTT GGT CAT CAC CA-3. The positive clones containing DNA inserts were linearized with Pme I and transformed into BJ5183-AD-1 competent cells to perform homologous recombination in *Escherichia coli* between the shuttle vectors carrying SUR2A gene and a large adenovirus containing plasmid following electroporation. Recombinants were identified from single colonies, linearized, and then transfected into HEK293 cells to produce infective adenovirus virions. Adenoviral particles were obtained by cell extraction after 7–10 days of transfection, and the primary virus was further amplified by infection of AD-293 cultures, amplified virus stock is prepared by 4 rounds of freeze/thaw. The virus titer is determined using QuickTiter Adenovirus Titer Immunoassay Kit (Cell Biolabs, Inc) according to the manufacturer's instructions. To infect H9C2 cells, a solution of recombinant adenovirus was mixed with culture medium, and cells were exposed to the virus with a multiplicity of 10 viral particles/cell for 48 h.

### 2.2 Real time RT-PCR

Total RNA was extracted from heart of rat H9C2 cells using TRIZOL reagent (Invitrogen, Carlsbad, CA) according to the manufacturer's recommendations. Extracted RNA was further purified by RNeasy Plus Mini Kit (Qiagen, Crawley, UK) according to the manufacturer's instructions. Rat primers for all K_ATP_ channel subunits were designed as depicted on Table 1. All K_ATP_ channel subunits were measured using these primers apart on SUR2A that was measured by combining SUR2A rat (Table 1) and mouse (sense: ACTATGGAGTCCGAGAACTA, antisense: AGGTTTGGACCAGTATCACA) primers. The structural similarity between mouse and rat SUR2A is 93%, but the primers used for mouse SUR2A do not recognize RAT SUR2A and *vice* *versa*. Therefore, we have used mouse SUR2A to make adenoviral construct, so that we can examine the level of adenoviral SUR2A expression in host H9C2 cells. To measure total SUR2A mRNA levels we have combined both sets of primers. The reverse transcription (RT) reaction was carried out with ImProm-II Reverse Transcriptase (Promega, Southampton, UK). A final volume of 20 µl of RT reaction containing 4 µl of 5× buffer, 3 mM MgCl_2_, 20 U of RNasin® Ribonuclease inhibitor, 1 U of ImProm-II reverse transcriptase, 0.5 mM each of dATP, dCTP, dGTP, and dTTP, 0.5 µg of oligo(dT), and 1 µg of RNA was incubated at 42 °C for 1 h and then inactivated at 70 °C for 15 min. The resulting cDNA was used as a template for real time PCR. A SYBR Green I system was used for the RT-PCR and the 25 µl reaction mixture contained: 12.5 µl of iQ™ SYBR® Green Supermix (2×), 7.5 nM each primers, 9 µl of ddH_2_O, and 2 µl of cDNA. In principle, the thermal cycling conditions were as follows: an initial denaturation at 95 °C for 3 min, followed by 40 cycles of 10 s of denaturing at 95 °C, 15 s of annealing at 56 °C, and 30 s of extension at 72 °C. The real time PCR was performed in the same wells of a 96-well plate in the iCycler iQ™ Multicolor Real-Time Detection System (Bio-Rad, Hercules, CA). Data was collected following each cycle and displayed graphically (iCycler iQ™ Real-Time Detection System Software, version 3.0A, Bio-Rad, Hercules, CA). Primers were tested for their ability to produce no signal in negative controls by dimer formation and then with regard to the efficiency of the PCR reaction. Efficiency is evaluated by the slope of the regression curve obtained with several dilutions of the cDNA template. Melting curve analysis tested the specificity of primers. Threshold cycle values, PCR efficiency (examined by serially diluting the template cDNA and performing PCR under these conditions) and PCR specificity (by constructing the melting curve) were determined by the same software. Each mouse cDNA sample was measured at three different quantities (and duplicated at each concentration, the corresponding no-RT mRNA sample was included as a negative control (blank). To determine relative mRNA expression (normalised to the wild type) we have used glyceraldehyde 3-phosphate dehydrogenase (GAPDH) as a control gene. The calculation of relative mRNA expression was performed as described [11]. The relative expression ratio (*R*) of SUR2A is calculated using equation *R* = (*E*_K_)^ΔCP_K_(WT-TG)^/(*E*_R_)^ΔCP_R_(WT-TG)^ where *E*_K_ is the real time PCR efficiency of SUR2A gene transcript, *E*_R_ is the real time PCR efficiency of GAPDH gene, ΔCP_K_ is the crossing point deviation of wild type-transgene (infected) of SUR2A gene transcript while ΔCP_R_ is the crossing point deviation of wild type-transgene of GAPDH gene transcript.

### 2.3 Patch clamp electrophysiology

To monitor whole cell K^+^ current the gigaohm seal patch clamp technique was applied in the perforated-patch whole cell configuration. H9C2 cells were superfused with Tyrode solution (in mM: 136.5 NaCl; 5.4 KCl; 1.8 CaCl_2_; 0.53 MgCl_2_; 5.5 glucose; 5.5 HEPES-NaOH; pH 7.4). All pipettes (resistance 3–5 MΩ), were filled with (in mM): KCl 140, MgCl_2_ 1, HEPES-KOH 5 and amphotericin B (Sigma, 240 μg/ml) (pH 7.3). For all cells monitored, the membrane potential was normally held at − 40 mV and the currents evoked by a series of 400 ms depolarising and hyperpolarising current steps (− 100 mV to + 80 mV in 20 mV steps) recorded directly to hard disk using an Axopatch-200B amplifier, Digidata-1321 interface and pClamp8 software (Axon Instruments, Inc., Forster City, CA). The capacitance compensation was adjusted to null the additional whole cell capacitative current. The slow capacitance component measured by this procedure was used as an approximation of the cell surface area and allowed normalisation of current amplitude (i.e. current density). Currents were low pass filtered at 2 kHz and sampled at 100 μs intervals.

### 2.4 Cell survival assay

The survival of H9C2 cells was assayed using Multitox-Fluor Multiplex Cytotoxicity Assay (Promega). Briefly, H9C2 cells were plated in complete media (DMEM containing 10% FCS) in 96-well plate, the recombinant adenovirus (GFP as a control or other adenoviruses as stated in the Results section) was added to the wells at the multiplicity of infection of 10. After a 48 h infection, the DNP was added to each well at the final concentration of 10 mM. To measure cell survival 6 h later, the peptide substrate (GF-AFC) that can be cleaved only by live cells was added to the each well. Following a 30 min-long incubation at 37 °C, plates were measured using 1420 Multibabel Counter (Victor) plate reader, with excitation at 370 nm and emissions of 480 nm. The percentage of live cells was calculated based on the intensity of fluorescence according to the manufacturer's instructions.

### 2.5 Luciferase assay

H9C2 cells were co-infected with adenoviruses containing genes encoding luciferase (to measure total ATP) or annexin-luciferase (to measure subsarcolemmal ATP) together with adenoviruses containing genes encoding K_ATP_ channel-forming proteins (as named in the Results section) 48 h before luciferase assay. To measure luciferase luminescence cells were mounted in 96-well plate in buffer with the following composition (in mM): 30 HEPES, 3 ATP, 15 MgSO_4_, 10 DTT; pH: 7.4. Some of the cells were untreated while the others were treated with 10 mM DNP. The reaction for luciferase luminescence measurement was initiated by adding 100 μM of luciferin and the luminescence was measured on a plate reader 1420 Multibabel Counter (Victor). Luminescence was measured in the absence of DNP and after 1 h of cell incubation with 10 mM DNP. To validate that annexin-luciferase- and luciferase-mediated signal reflect subsarcolemmal and cytosolic ATP respectively, cells were infected with either luciferase, annexin-luciferase or GFP (control), harvested after 48 h and plasma membrane and cytosolic fractions were obtained as described in [7]. The presence/absence of plasma membrane in a fraction was verified by 5-nucleotidase assay. Luminescence was measured as described above; in membrane and cytosolic fractions (both spiked with 5 mM ATP) of cells infected with annexin-luciferase the luminescence signal was 6956 AU/mg protein and 480 AU/mg protein respectively. These results demonstrated that targeted expression of luciferase to membrane was successful and that signal *in vivo* reflects mostly the level of ATP in environment surrounding the plasma membrane. On the other hand, in cells infected with luciferase, luminescence was 1030 AU/mg protein in cytosolic fraction and 670 AU/mg protein in membrane fraction showing ubiquitous distribution of luciferase.

### 2.6 Immunoprecipitation/Western blotting

The cells were homogenised in buffer (TRIS 10 mM, NaH_2_PO_4_ 20 mM, EDTA 1 mM, PMSF 0.1 mM, pepstatin 10 μg/ml, leupeptin 10 μg/ml, at pH = 7.8) and centrifugated at 500 *g* (to remove large particles from the homogenate). To obtain cellular membrane fraction cells were homogenised in buffer I (TRIS 10 mM, NaH_2_PO_4_ 20 mM, EDTA 1 mM, PMSF 0.1 mM, pepstatin 10 μg/ml, leupeptin 10 μg/ml, at pH = 7.8) and incubated for 20 min (at 4 °C). The osmolarity was restored with KCl, NaCl and sucrose and the obtained mixture was centrifugated at 500 *g*. The supernatant was diluted in buffer II (imidazole 30 mM, KCl 120 mM, NaCl 30 mM, NaH_2_PO_4_ 20 mM, sucrose 250 mM, pepstatin 10 μg/ml, leupeptin 10 μg/ml, at pH = 6.8) and centrifugated at 7000 *g*, pellet removed and supernatant centrifugated at 30 000 *g*. The obtained pellet contains membrane fraction. Protein concentration was determined using the method of Bradford; 10 µg of the anti-Kir6.2 antibody was prebound to Protein-G Sepharose beads and used to immunoprecipitate from 50 µg of membrane fraction protein extract. The pellets of this precipitation were run on SDS-polyacrylamide gels for Western analysis. Western blot probing was performed using 1/1000 dilution of anti-SUR2 or anti-LDH antibody, respectively, and detection was achieved using Protein-G HRP and ECL reagents. The band intensities were analysed using the Quantiscan software.

### 2.7 Statistical analysis

Data are presented as mean ± SEM, with *n* representing the number of independent experiments. Mean values were compared by the ANOVA followed by Student's *t*-test or by Student's *t*-test alone where appropriate using SigmaStat program (Jandel Scientific, Chicago, Illinois). *P* < 0.05 was considered statistically significant.

## 3 Results

### 3.1 Infection with SUR2A increases the level of SUR2A in H9C2 cells

**Fig. 1:**
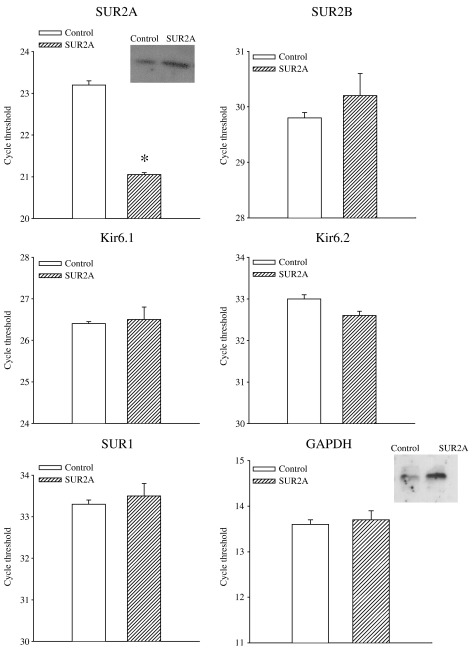
Infection with AV-SUR2A increases expression of SUR2A without affecting the expression of other K_ATP_ channel subunits. Bar graphs represent cycling thresholds of the real time RT-PCR progress curves of K_ATP_ channel-forming subunits. Inset in SUR2A graph is a Western blot of anti-Kir6.2 immunoprecipitate from H9C2 cells with either anti-SUR2 or anti-GAPDH antibody under depicted conditions (for SUR2 the signal depicted was between 97 and 191 kDa while for GAPDH it was ∼ 39 kDa as determined by SeeBlue Plus Prestained Standard, Invitrogen; similar results were obtained in three independent experiments). Each bar represents mean ± SEM (*n* = 4 for each). *P < 0.05.

We have analysed levels of SUR2A mRNA in control cells and cells infected with AV-SUR2A using real time RT-PCR. This method has revealed that infection with AV-SUR2A has significantly increased mRNA levels of SUR2A (cycling threshold was 23.20 ± 0.10 in control cells and 21.05 ± 0.05 in infected cells, *n* = 4 for each, *P* < 0.01, Fig. 1). No statistically significant difference was observed in mRNA levels of other K_ATP_ channel-forming subunits (Fig. 1). It is estimated that infected cells had 4.22 times more SUR2A mRNA than the control cells. Furthermore, we have probed anti-Kir6.2 immunoprecipitate of H9C2 extract with anti-SUR2 antibody. Using this strategy, we measured only those Kir6.2 and SUR2A subunits that physically assemble in sarcolemma to form a channel. Western blot has demonstrated that the level of myocardial SUR2A protein was increased in H9C2 cells in comparison to control cells (Fig. 1). Another protein known to be a part of the sarcolemmal K_ATP_ channel protein complex, GAPDH [12], was also increased in cell infected with SUR2A (Fig. 1).

**Fig. 2:**
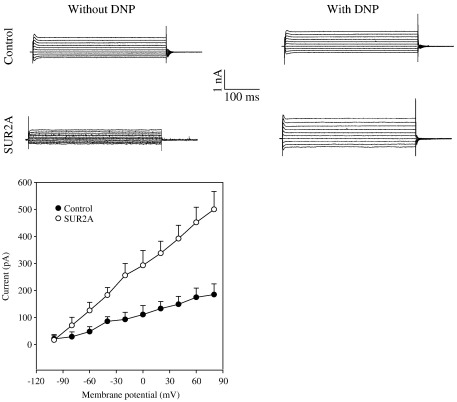
Infection with AV-SUR2A increases DNP-induced whole cell K^+^ current. Original membrane currents under depicted conditions (DNP concentration was 10 mM) and line-scatter graph showing DNP-induced K^+^ current component (obtained by substraction of whole cell K^+^ current in the presence and absence of DNP) in control cells (control) and cells infected with AV-SUR2A (SUR2A). Each point represents mean ± SEM (*n* = 5 for each).

It is well established that K_ATP_ channels are activated when cardiac cells are challenged with 2.4-dinitrophenol (DNP). This channel activation is cellular self-protective mechanism as it hyperpolarize the membrane, inhibits influx of Ca^2+^ and counteracts DNP-induced cellular damage [13–16]. We have applied perforated patch clamp electrophysiology to test whether infection with AV-SUR2A has any effect on DNP-induced whole cell K^+^ current. Perforated patch whole cell recording does not impair intracellular milieu [17], which allows monitoring the behaviour of K_ATP_ channels-conducted K^+^ current during stress under conditions of intact intracellular environment. In control cells, exposure to DNP (10 mM) has induced a significant increase in whole cell K^+^ current flowed through K_ATP_ channels [see also 4,5], but this was more pronounced in cells infected with SUR2A (Fig. 2).

### 3.2 Infection with SUR2A increase cellular resistance to severe metabolic stress

**Fig. 3:**
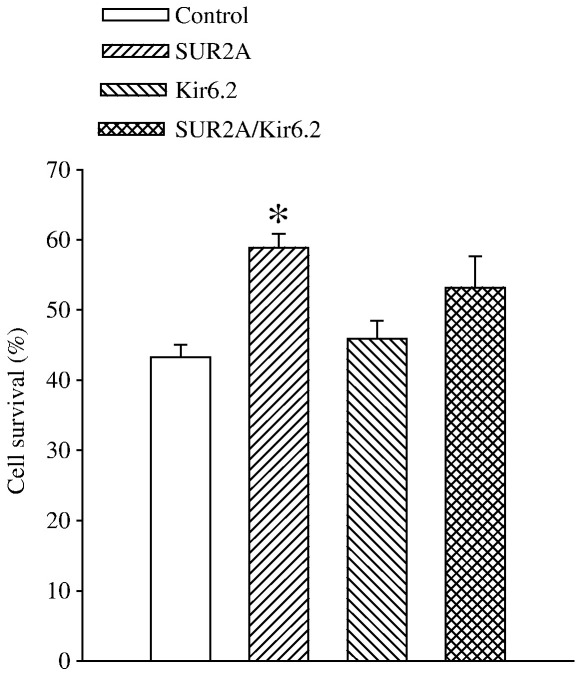
Infection with AV-SUR2A increases survival of cells exposed to DNP. A bar graph showing a percentage of survival in control cells and cells infected with SUR2A alone, Kir6.2 alone or SUR2A/Kir6.2 exposed to DNP (10 mM). Each bar represent mean ± SEM (*n* = 3–14). *P < 0.05 when compared to control.

DNP is known metabolic inhibitor that is used to induce metabolic stress in different cell types [18]. When control cells were treated with DNP (10 mM), only 43.3 ± 1.8% (*n* = 14) of cells have survived this insult (Fig. 3). Cells infected with SUR2A were significantly more resistant to DNP (10 mM) than control cells (58.9 ± 2.0% of cells infected with SUR2A have survived DNP, *n* = 8, *P* < 0.001 when compared to the control, Fig. 3). On the other hand, infection with Kir6.2 alone did not have any effect on cellular survival under DNP (10 mM; 45.9% ± 2.6%, *n* = 3, *P* = 0.93 when compared to the control cells, Fig. 3) neither infection with SUR2A/Kir6.2 improved the effect of SUR2A alone (53.2% ± 4.1%, *n* = 3, *P* = 0.28 when compared with cells infected with SUR2A alone, Fig. 3).

### 3.3 Infection with Kir6.2AFA abolishes DNP-induced K^+^ current in control and cells infected with SUR2A

**Fig. 4:**
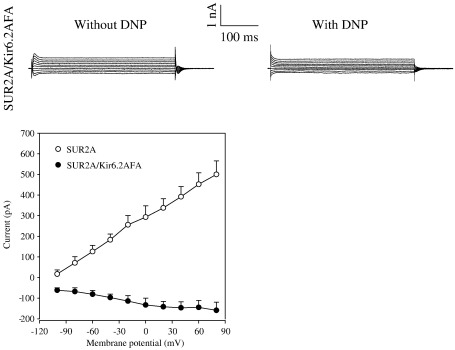
Kir6.2AFA abolishes SUR2A-mediated increase in K^+^ current. Original membrane currents under depicted conditions (DNP concentration was 10 mM) and line-scatter graph showing DNP-induced K^+^ current component (obtained by substraction of whole cell K^+^ current in the presence and absence of DNP) in cells infected with AV-SUR2A (SUR2A) and AV-SUR2A plus AV-Kir6.2AFA. Each point represents mean ± SEM (*n* = 4 for each).

Kir6.2AFA is a mutant Kir6.2 form with largely reduced ability to conduct K^+^. When Kir6.2AFA is introduced into H9C2 cells DNP does not induce increase in K^+^ current, which is in agreement with notion that treatment with DNP activates sarcolemmal K_ATP_ channels [4,5]. In cells infected with SUR2A/Kir6.2AFA DNP did not induce increase in whole cell K^+^ current (Fig. 4).

### 3.4 Kir6.2AFA only partially reduces the SUR2A-mediated cytoprotective effect

**Fig. 5:**
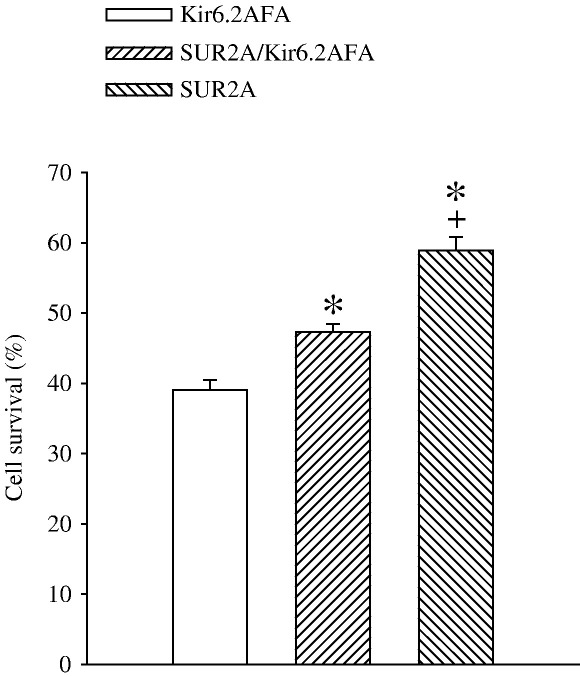
Kir6.2AFA only partially inhibits SUR2A-mediated cytoprotection. A bar graph showing a percentage of survival in cells infected with Kir6.2AFA alone, Kir6.2AFA/SUR2A and SUR2A alone exposed to DNP (10 mM). Each bar represent mean ± SEM (*n* = 5–14). *P < 0.05 when compared to the control.

In cells infected with Kir6.2AFA alone, survival rate in 10 mM DNP-treated cells was 39.1 ± 1.4% (*n* = 6) and that was slightly, but significantly, lower than survival of the control cells (43.3 ± 1.8%, *n* = 14; Fig. 5). A concomitant infection of H9C2 cells with SUR2A and Kir6.2AFA has resulted in cell survival of 47.3 ± 1.2% (*n* = 5), which was significantly lower than survival in cells infected with SUR2A (58.9 ± 2.0%, *n* = 8, *P* < 0.01), but significantly higher than survival in cells infected with Kir6.2AFA alone (47.3 ± 1.2% with Kir6.2AFA/SUR2A *n* = 5, *P* < 0.001 when compared to Kir6.2AFA cells, Fig. 5).

### 3.5 Infection with SUR2A increases levels of subsarcolemmal ATP in H9C2 cells

**Fig. 6:**
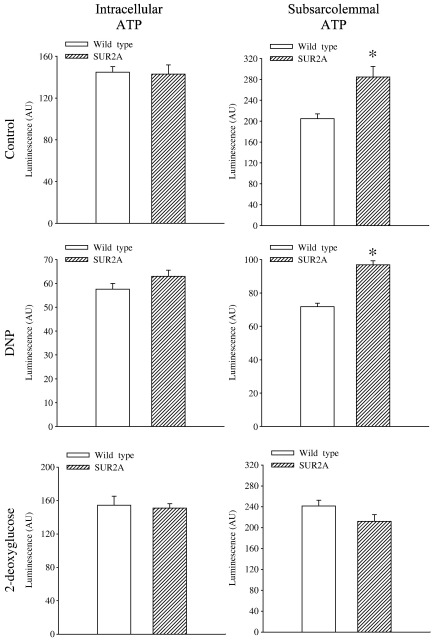
Infection with AV-SUR2A increases the levels of subsarcolemmal ATP. Bar graphs showing luciferase (intracellular ATP) and annexin-luciferase (subsarcolemmal ATP) luminescence in control cells and cells infected with AV-SUR2A under control conditions (control) and after treatment with either 10 mM DNP (DNP) or 50 mM 2-deoxyglucose (2-deoxyglucose). Each bar represent mean ± SEM (*n* = 5). *P < 0.05.

Sarcolemmal K_ATP_ channel complex seems to contain, besides the channel subunits, glycolytic and other enzymes that catalyses reactions producing ATP [9,12,19–22]. Anything that would increase the levels of ATP would improve cellular well being under different conditions of stress. Therefore, we have tested the levels of intracellular and subsarcolemmal ATP using luciferase and annexin-luciferase constructs respectively. Infection of SUR2A did not significantly change intracellular level of ATP (the intensity of luciferase luminescence was 144.8 ± 5.3 AU in control and 142.8 ± 8.9 AU in SUR2A cells, *n* = 5, *P* = 0.85, Fig. 6). However, infection with SUR2A did increase subsarcolemmal levels of ATP (the intensity of annexin-luciferase luminescence was 204.8 ± 9.0 AU in control and 284.6 ± 20.4 AU in SUR2A cells, *n* = 5, *P* = 0.01, Fig. 6). After treatment with DNP (10 mM) total intracellular ATP levels were not different in control cells and cells infected with SUR2A (the intensity of luciferase luminescence was 57.6 ± 2.4 AU in control cells and 63.0 ± 2.5 AU in SUR2A cells, *n* = 5, *P* = 0.16, Fig. 6). In contrast, subsarcolemmal ATP levels were significantly increased in SUR2A-infected cells (the intensity of annexin-luciferase luminescence was 71.8 ± 2.1 AU in control cells and 96.8 ± 2.4 AU in SUR2A cells, *n* = 5, *P* < 0.001, Fig. 6).

An increased ATP levels in subsarcolemmal space would be in accord with the idea that enzymes physically associated with the K_ATP_ channel subunits produce ATP and that infection with SUR2A increases number of ATP-producing K_ATP_ channel protein complexes [4,5]. So far, five glycolytic enzymes were identified as parts of sarcolemmal K_ATP_ channel protein complex [9,10,22]. Here, to test whether SUR2A-mediated increase in subsarcolemmal ATP is associated with glycolysis, we have examine the effect of 2-deoxyglucose on SUR2A-mediated increase in cytosolic and subsarcolemmal ATP. 2-deoxyglucose (50 mM) blocked the effect of SUR2A on subsarcolemmal ATP (the intensity of annexin-luciferase luminescence was 241.6 ± 11.1 AU in control cells and 212.2 ± 12.8 AU in SUR2A cells, *n* = 5, *P* = 0.12, Fig. 6). SUR2A also did not affect intracellular ATP levels in cells treated by 2-deoxyglucose (50 mM; the intensity of luciferase luminescence was 154.4 ± 10.8 AU in control cells and 151.0 ± 5.2 AU in SUR2A cells, *n* = 5, *P* = 0.78, Fig. 6).

### 3.6 Expression of Kir6.2AFA does not modify effect of SUR2A on ATP levels

**Fig. 7:**
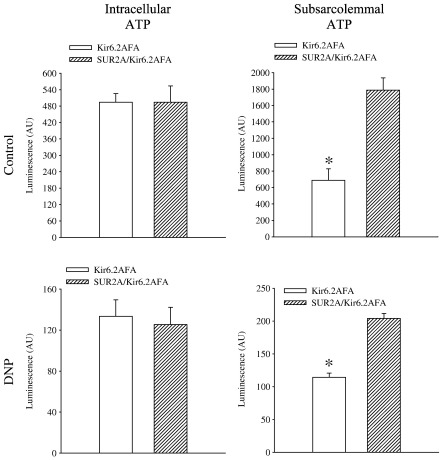
Kir6.2AFA does not modify the effect of AV-SUR2A on ATP levels. Bar graphs showing luciferase (intracellular ATP) and annexin-luciferase (subsarcolemmal ATP) luminescence in cells infected with kir6.2AFA alone and SUR2A/Kir6.2AFA under control conditions (control) and after treatment with 10 mM DNP (DNP). Each bar represent mean ± SEM (*n* = 5). *P < 0.05.

It is already known that infection with Kir6.2AFA *per se* does not affect either total or subsarcolemmal ATP levels [4]. When Kir6.2AFA cells were infected with SUR2A no changes in intracellular ATP was observed (the intensity of luminescence was 494.0 ± 31.2 AU in Kir6.2AFA and 493.6 ± 59.7 AU in Kir6.2AFA/SUR2A cells, *n* = 5, *P* = 0.99, Fig. 7). However, infection with SUR2A has significantly increased the levels of subsarcolemmal ATP (the intensity of luminescence was 688.2 ± 140.7 AU in Kir6.2AFA and 1787.2 ± 148.9 AU in Kir6.2AFA/SUR2A cells, *n* = 5, *P* < 0.001, Fig. 7). After treatment with DNP (10 mM), intracellular ATP levels were also not different between the cellular phenotypes (the intensity of luminescence was 133.4 ± 16.1 AU in Kir6.2AFA and 125.4 ± 16.9 AU in Kir6.2AFA/SUR2A cells, *n* = 5, *P* = 0.74, Fig. 7), as opposed to the level of subsarcolemmal ATP that was significantly increased in cells infected with SUR2A (the intensity of luminescence was 114.3 ± 6.5 AU in Kir6.2AFA and 204.0 ± 7.6 AU in Kir6.2AFA/SUR2A cells, *n* = 5, *P* < 0.001, Fig. 7).

### 3.7 193glyM-LDH inhibits the effect of SUR2A on subsarcolemmal ATP and DNP-induced K^+^ current

**Fig. 8:**
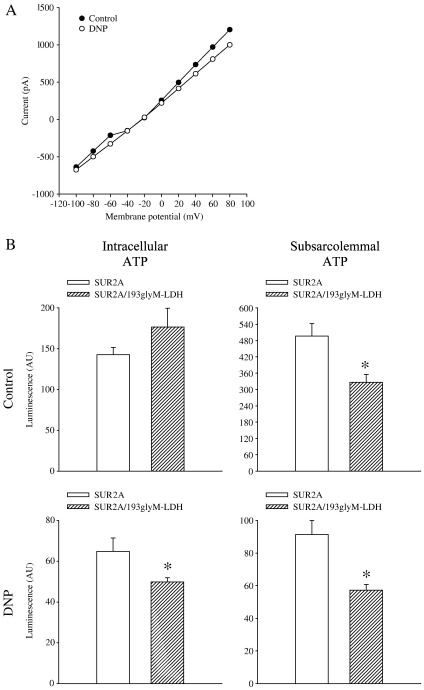
193gly-M-LDH inhibits effects of SUR2A on DNP-induced K^+^ current and ATP levels. A. Line-scatter graph showing DNP-induced K^+^ current component in cells infected with AV-193gly-M-LDH in the absence (control) and presence of DNP (10 mM). Each point represents mean (*n* = 4). B. Bar graphs showing luciferase (intracellular ATP) and annexin-luciferase (subsarcolemmal ATP) luminescence in cells infected with SUR2A alone and SUR2A/193gly-M-LDH under control conditions (control) and after treatment with 10 mM DNP (DNP). Each bar represent mean ± SEM (*n* = 5). *P < 0.05.

We have recently shown that 193glyM-LDH, a mutant form of muscle lactate dehydrogenase (M-LDH) that has lost its catalytic activity while retained the ability to physically interact with Kir6.2/SUR2A, inhibits both DNP-induced K_ATP_ channel activation and ATP production by sarcolemmal K_ATP_ channels [4]. In cells infected with both SUR2A and 193glyM-LDH, DNP did not increase whole cell K^+^ current (Fig. 8A). The luciferase luminescence in cells infected with both SUR2A and 193glyM-LDH was 176.6 ± 22.8 AU (*n* = 5) and that was not significantly different from those infected with SUR2A alone where luciferase fluorescence was 142.8 ± 8.9 AU (*n* = 5, *P* = 0.23 when compared to SUR2A/193glyM-LDH cells, Fig. 8B). The annexin-luciferase fluorescence was 326.8 ± 28.1 AU (*n* = 5) in SUR2A/193glyM-LDH cells which was significantly lower than those in SUR2A cells (annexin-luciferase fluorescence was 496.4 ± 45.8 in SUR2A cells, *P* = 0.01 when compared to SUR2A/193glyM-LDH cells, *n* = 5, Fig. 8B). Similar results were obtained when ATP levels were measured after treatment with DNP (10 mM). For cells infected with SUR2A alone, luciferase and annexin-luciferase fluorescence were 64.8 ± 6.6 AU and 91.4 ± 9.9 AU respectively (*n* = 5 for all; Fig. 8B). Both of these values were significantly higher than in cells infected with SUR2A/193glyM-LDH (luciferase: 49.8 ± 2.1 AU, *n* = 5, *P* < 0.001 when compared to SUR2A cells; annexin-luciferase: 57.2 ± 3.6 AU, *n* = 5, *P* < 0.001 when compared to SUR2A cells, Fig. 8B).

### 3.8 193glyM-LDH inhibits SUR2A-mediated cytoprotection

**Fig. 9:**
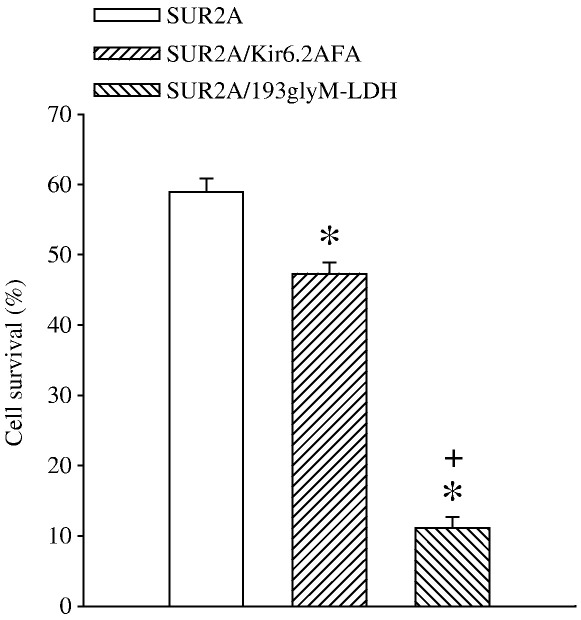
Infection with AV-SUR2A increases survival of cells exposed to DNP. A bar graph showing a percentage of survival in cells infected with SUR2A alone, SUR2A/Kir6.2AFA and SUR2A/193gly-M-LDH exposed to DNP (10 mM). Each bar represent mean ± SEM (*n* = 3–6). *P < 0.05 when compared to SUR2A and ^+^*P* < 0.05 when compared to SUR2A/Kir6.2AFA.

193glyM-LDH has shown to simultaneously inhibit both mechanisms that could contribute to the SUR2A-mediated cytoprotection (increase in subsarcolemmal ATP and increase in K^+^ current). Therefore, we have assessed the effect that 193glyM-LDH has on SUR2A-mediated cytoprotection. Cells co-infected with SUR2A/193glyM-LDH were significantly more sensitive to DNP (10 mM) than cells infected with SUR2A alone (cell survival was 11.1 ± 1.6% for SUR2A/193glyM-LDH cells and 58.3 ± 1.6% for SUR2A cells, *n* = 3–6, *P* < 0.001, Fig. 9) and cells co-infected with SUR2A/Kir6.2AFA (cell survival was 47.3 ± 1.6% in these cells, *n* = 6, *P* < 0.001 when compared to SUR2A/193glyM-LDH cells).

## 4 Discussion

In the present study we have shown that SUR2A mediate cardioprotection by increasing the numbers of sarcolemmal K_ATP_ channels and consequent increase in K^+^ current and subsarcolemmal ATP levels.

It has been previously shown that moderate increase in SUR2A generates a myocardial phenotype with increased numbers of sarcolemmal K_ATP_ channels and increased resistance to ischaemia–reperfusion [2]. In this particular phenotype SUR2A expression was under control of CMV promoter, which is the promoter used in AV-SUR2A in this study as well. Under CMV promoter SUR2A mRNA in hearts of transgenic animals was increased for ∼ 6 times in comparison to the wild type [2], which is similar to those in H9C2 cells achieved by AV-SUR2A. This modest up-regulation of SUR2A has resulted in increased number of sarcolemmal K_ATP_ channels, which is in accord to what has been observed in transgenic CMV-SUR2A phenotype [2]. It should be mentioned that a transgenic phenotype overexpressing SUR2A by action of αMHC promoter has decreased the number of sarcolemmal K_ATP_ channels [23]. However, as opposed to infected H9C2 cells, in the phenotype described by Flagg et al. mRNA SUR2A levels were increased by ∼ 50 times. It has been reported that cardiac Kir6.2 mRNA levels are physiologically ∼ 30 times higher than those values of SUR2A [2]. How this transpose into difference at protein level is not yet known, but it is likely that some difference between protein levels also exist. It is therefore possible that stimulating expression of SUR2A over the levels of Kir6.2 expression would suppress the functional expression of K_ATP_ channels from optimized dimeric SUR2A/Kir6.2. A similar idea about the regulation of K_ATP_ channel numbers has been put forward by Flagg et al. [23] and this could explain why a moderate, but not extreme, increase in SUR2A levels results in increased number of sarcolemmal K_ATP_ channels.

DNP is a known metabolic inhibitor that was used in many studies to induce metabolic stress in different cell types [18]. When applied, this compound induces chemical hypoxia, which activates K_ATP_ channels [24]. In turn, such activation of the channels decreases a degree of cell injury [13]. The finding that DNP-induced K^+^ current was increased in cells infected with SUR2A is in accord with these cells having increased number of sarcolemmal K_ATP_ channels. An increase in sarcolemmal K_ATP_ channels is associated with increased myocardial resistance to metabolic stress [7,8,25]. However, there is a report suggesting that a lack of SUR2A, but not Kir6.2 [26], generates a cardiac phenotype with increased resistance to ischaemia/reperfusion [27]. Most recently, this has been explained by the presence of a short SUR2A form in mitochondria when it plays a cardioprotective role that remained unaffected in SUR2A knockout phenotype [28]. On the other hand, transgenic mice with moderately increased expression of SUR2A also acquire resistance to metabolic stress, which was explained by increase in numbers of sarcolemmal K_ATP_ channels and earlier channel activation in response to metabolic stress [2]. Here, we have shown that an acute increase in SUR2A expression confers cytoprotection, which would be in accord with reports associating increased SUR2A with the cardioprotective outcome [29].

The mechanism of cytoprotection afforded by K_ATP_ channels is a long-standing issue. It is predominant view that the activation of the channel is required for K_ATP_ channels to protect the heart against metabolic stress [30]. How opening of sarcolemmal K_ATP_ channels protect the heart against metabolic stress is still to be fully understood. It is traditional view that the activation of K_ATP_ channels shortens action membrane potential resulting in decreased influx of Ca^2+^ and prevention of Ca^2+^ overload [30]. However, it has been shown that the activation of K_ATP_ channels is protective in cells that do not generate action membrane potential, including diastolic cardiomyocytes [14–16]. More recently, we have suggested that cytoprotection afforded by K_ATP_ channels could also involve a channel activity-independent mechanism [4,5]. Specifically, it has been found that inhibition of the catalytic activity of M-LDH, an enzyme physically associated with sarcolemmal K_ATP_ channels [9], results in decreased ATP levels as well as inhibited K_ATP_ channels-mediated K^+^ current in response to stress. The inhibition of both the channel activity and ATP production had much more pronounced effect on cell survival than a sole inhibition of channel activity [4,5]. Based on these findings, it was concluded that production of ATP by sarcolemmal K_ATP_ channel protein complex is a significant part of the mechanism underlying K_ATP_ channel-mediated cardioprotection. In the present study, we have shown that increase in SUR2A expression increases subsarcolemmal ATP levels even under control conditions. This would support the notion that sarcolemmal K_ATP_ channel protein complex produces ATP and that this property could be important even under physiological conditions. The structural studies of K_ATP_ channels have shown that channel subunits *in vivo* are physically associated with enzymes that catalyses ATP production, most of them being involved in glycolysis [9,12,22]. Such composition of sarcolemmal K_ATP_ channels would allow a supposition that K_ATP_ channel protein complex could serve as ATP-producing machinery in the sarcolemma. To test hypothesis that production of ATP by glycolytic enzymes physically associated with K_ATP_ channel subunits mediates the effect of SUR2A on subsarcolemmal ATP, we have used 2-deoxyglucose, a known inhibitor of glycolysis. Treatment with this compound abolished the effect of SUR2A on subsarcolemmal ATP showing that glycolysis is essential for the observed SUR2A effect. This would be in agreement with the idea that ATP produced by glycolytic enzymes physically associated with K_ATP_ channel subunits underlies SUR2A-mediated increase in subsarcolemmal ATP levels. The fact that SUR2A counteracted DNP-induced loss of subsarcolemmal ATP further supports our hypothesis that fully assembled K_ATP_ channel protein complexes might serve as an ATP producer *in vivo*. It would be logical to expect that more subsarcolemmal ATP would support ion homeostasis by providing energy for active transport processes.

As opposed to Kir6.2AFA that inhibits K_ATP_ channels permeability without affecting subsarcolemmal ATP levels, 193glyM-LDH has effects on both the channel activity and subsarcolemmal ATP [4]. Lactate dehydrogenase (LDH) is a tetramer composed of either M (muscle) and/or H (heart) subunits, which may be combined to form five LDH isozymes. In the heart, all LDH isozymes are present; LDH_1_ (H4) is the predominant form, whereas LDH_5_ (M4) is present in trace amounts [31]. It has been shown that M-LDH, but not H-LDH, physically associates with Kir6.2 and SUR2A subunits and is integral part of the sarcolemmal K_ATP_ channel protein complex [9]. DNP is a known inhibitor of oxidative phosphorylation, but the mechanism of DNP-induced activation of sarcolemmal K_ATP_ channels is less associated with decrease of intracellular ATP and more with increase in intracellular lactate levels and acidification [32–35]. It seems that lactate directly targets and activates K_ATP_ channels despite the presence of high intracellular levels of ATP while low pH counteract the channel inhibition induced by ATP [36]. It has been demonstrated, at the both recombinant and native levels, that M-LDH physically associated with K_ATP_ channels is required, by virtue of its catalytic activity, for the channel opening during metabolic stress [4,5,9]. It has been suggested that M-LDH-produced lactate 1) targets and activates K_ATP_ channels and 2) decreases the channel sensitivity to inhibitory effect of ATP [36,37]. At the same time M-LDH activity contributes to the ATP production. The expression of 193glyM-LDH, a catalytically inactive form of M-LDH, has been shown to inhibit both M-LDH properties to activate K_ATP_ channels (by producing lactate) and counteract DNP-induced decrease in subsarcolemmal ATP. Here, we have shown that infection with 193glyM-LDH abolishes SUR2A-mediated increase in K^+^ current as well as increase in subsarcolemmal ATP levels. This finding is in accord with our hypothesis that infection with AV-SUR2A has increased the number of sarcolemmal K_ATP_ channel protein complexes that are both important for subsarcolemmal ATP levels and K^+^ current in response to metabolic stress. An increase in subsarcolemmal ATP and K_ATP_ channel activation might look as a contradiction. However, it is known that ischaemia opens sarcolemmal K_ATP_ channels before the fall of intracellular ATP occur [38], suggesting that the intracellular level of ATP is not the only factor that regulates the channel activity. In addition to ATP, it has been suggested that the activity of these channels may be regulated by other nucleotides, intracellular pH, lactate, cytoskeleton, protein kinase C, phosphatidylinositol-4,5-bisphosphate, and by the operative condition of the channel itself [39]. It is well established that lactate activates K_ATP_ channels in the presence of millimolar ATP, though the mechanism of this activation is yet unknown [9]. Therefore, it is quite possible that M-LDH catalytic activity results in both increase in subsarcolemmal ATP (the effect of ATP production) and K_ATP_ channel activity (the effect of lactate production). How significant is increase in subsarcolemmal ATP for SUR2A-mediated cell survival was assessed by comparing the effect of DNP in cell expressing SUR2A/Kir6.2AFA (inhibited channel activity without changes in subsarcolemmal ATP) and cells expressing SUR2A/193glyM-LDH (inhibited channel activity as well as increase in subsarcolemmal ATP). It has been found that, unlike Kir6.2AFA, expression of 193glyM-LDH abolished cytoprotection induced by SUR2A and had a deleterious effect on cell survival under DNP. The fact that 193glyM-LDH was more efficient in inhibiting SUR2A than Kir6.2AFA supports our hypothesis that infection of H9C2 cells with AV-SUR2A led to increased numbers of sarcolemmal K_ATP_ channels, increase in subsarcolemmal ATP and increased K^+^ current in response to metabolic stress. An increase in subsarcolemmal ATP following infection with SUR2A seems to be the major mechanism of SUR2A-mediated cardioprotection. It is also worthwhile mentioning that cell survival after concomitant infection with SUR2A and Kir6.2 was not statistically different from those in cells infected with SUR2A alone. This is compatible with idea that SUR2A level is the rate limiting factor in regulating numbers of sarcolemmal K_ATP_ channels and cell resistance to metabolic stress.

In conclusion, this study has shown that infection with SUR2A increases resistance to metabolic stress in H9C2 cells. The mechanism underlying SUR2A-mediated cytoprotection is associated with increase in number of sarcolemmal K_ATP_ channels, increased K^+^ current as well as increased production of ATP by the glycolytic enzymes physically associated with K_ATP_ channel subunits.

## Figures and Tables

**Table 1:** Rat primers used in real time RT-PCR experiments.

mRNA	Sense		Antisense		PCR product
SUR 1	5′-GGAAGGACTC ACCACCATC-3′	19 bp	5′-GAGACCATC AAGGCATAGG-3′	19 bp	252 bp
SUR2A	5′-ACTTCAGCGT TGGACAGAGAC-3′	21 bp	5′-AGCAGGTTTGG ACCAGTATCG-3′	21 bp	259 bp
SUR2B	5′-GACGCCA CTGTCACCGAAG-3′	19 bp	5′-TCATCACAATG ACCAGGTCAGC-3′	22 bp	244 bp for SUR2B 420 bp for SUR2A
Kir6.1	5′-GTCACACGCTG GTCATCTTCAC-3′	22 bp	5′-GGCACTCCTCAG TCATCATTCTCC-3′	24 bp	249 bp
Kir6.2	5′-TGGCTGACGAG ATTCTGTGG-3′	20 bp	5′-TGGCGGGGCTG TGCAGAG-3′	17 bp	130 bp
GAPDH	5′-ATAGAATTCC ATGACAAAGTGGAC ATTGTTGCCA-3′	34 bp	AGCCTCGAGTTA GGAAATGAG CTTCACAAAGTT-3′	33 bp	860 bp
